# Structural Lesion Progression of the Sacroiliac Joint and Clinical Features in axSpA During TNFi Reduction: A Retrospective Cohort Study

**DOI:** 10.3389/fmed.2021.781088

**Published:** 2021-12-07

**Authors:** Qian Mo, Yuanji Dong, Cong Ye, Jixin Zhong, Shaozhe Cai, Min Wang, Lingli Dong

**Affiliations:** ^1^Department of Rheumatology and Immunology, Tongji Hospital, Tongji Medical College, Huazhong University of Science and Technology, Wuhan, China; ^2^Department of Radiology, Tongji Hospital, Tongji Medical College, Huazhong University of Science and Technology, Wuhan, China

**Keywords:** axial spondyloarthritis, tumor necrosis factor inhibitor, tapering, sacroiliac joint, magnetic resonance imaging

## Abstract

**Objective:** In the clinic, some patients with axial spondyloarthritis (axSpA) have to reduce tumor necrosis factor inhibitor (TNFi) for various reasons. However, there are few studies about how to balance the relapse and TNFi reduction. Here we retrospectively analyzed the structural progression of the sacroiliac joint (SIJ) and clinical features in axSpA during TNFi reduction.

**Methods:** A total of 108 patients with axSpA who followed up for 2 years and completed at least baseline, 12-month, and 24-month MRI scans of SIJ were divided into the tapering group (*n* = 63) and withdrawal group (*n* = 45) according to whether TNFi was stopped. We divided 2 years into five intervals, calculating the average dose quotient (DQ) for each of 540 intervals from 108 patients. By using generalized estimation equations with inverse probability of treatment weighting, we investigated the unbiased effects of average DQ on structural progression and treatment response.

**Results:** The disease activity (such as Bath Ankylosing Spondylitis Disease Activity Index (BASDAI), Bath Ankylosing Spondylitis Functional Index (BASFI), Ankylosing Spondylitis Disease Activity Score (ASDAS)-CRP, and ASDAS-ESR) and relapse rate were lower in the tapering group at 12 and 24 months (*p* < 0.05). Δerosion (β = −0.0100, *p* = 0.00026) and Δthe Spondyloarthritis Research Consortium of Canada (SPARCC; β = −0.0959, *p* < 0.0001) were negatively correlated with average DQ. The average DQ 30 (74.8%, 80.0%) or 41.6 (76.5%, 83%) was best to discriminate the status of treatment response or the status of bone marrow edema, but considering operability, the average DQ 25 (78.0%, 63.3%) was also acceptable especially for patients with HLA-B27 negative and non-severe fat metaplasia.

**Conclusion:** Complete TNFi withdrawal was not recommended. Our study provided a referable strategy (tapering then maintained the average DQ over 30 or even 25) for patients who need TNFi reduction. Higher dose usage of TNFi was associated with a slower erosion progression of SIJ.

## Introduction

Axial spondyloarthritis (axSpA) is a chronic inflammatory disease that mainly affects the spine and sacroiliac joints (SIJ) (1–3). And the inflammatory response and structural damage can cause serious impairment in physical flexibility, work efficiency, and life quality (4–6). According to the radiologic sacroiliitis manifestations, it can be divided into non-radiographic axial spondyloarthritis (nr-axSpA) and radiographic axSpA, and the latter mainly includes ankylosing spondylitis (AS) (7–9). Although divided into two categories, patients with nr-axSpA or AS are similar in symptom burdens, clinical features, comorbidity, and tumor necrosis factor inhibitor (TNFi) response.

A series of randomized controlled trials and large cohort studies demonstrated that patients with axSpA responded well to the full dose of the TNFi treatment and most of these studies focused on the radiographic progression of the spine in patients with AS (10–20). Although the radiographic improvement effect on the spine is controversial, results from a meta-analysis published in 2020 indicated that >4 years of TNFi usage was associated with delayed structural progression of the spine (21). However, in the real world, it is inescapable that some patients have to opt for dose reduction or withdrawal because of intolerance to full-standard TNFi, risk of potential infection, unaffordability to high expense, intolerability for long-term subcutaneous injection, or multiple reasons (22, 23), while complete withdrawal often leads to relapse (24, 25). Several studies have begun to use tapering strategy for patients with axSpA and have shown that tapering TNFi to the extent of 75% and 50% full dose has comparable efficacy in maintaining low disease activity (26–32). The European Alliance of Associations for Rheumatology (EULAR) recently recommended that tapering TNFi can be considered for patients who have achieved sustained remission (2). Although the tapering strategy is recommended, there are limited studies focused on the referable strategy of tapering. In addition, previous studies mainly focused on the radiographic changes of the spine, while the impact of tapering TNFi on structural lesion progression of the SIJ was rarely studied. In this study, we retrospectively observed the effect of the TNFi tapering strategy on disease activity and the structural progression of the SIJ.

## Methods

### Study Population and Clinical Assessment

We retrospectively reviewed the medical data of 2,272 patients with axSpA who were admitted to Tongji Hospital from January 2017 to January 2021. The diagnosis was made according to the Assessment of Spondyloarthritis international Society (ASAS) classification criteria for axSpA or modified 1984 New York criteria for AS (7, 8). Among them, patients who had been treated with TNFi or other biological agents before the first visit or combined continuous treatment with nonsteroidal anti-inflammatory drugs (NSAIDs) or disease-modifying antirheumatic drugs (DMARDs) were excluded. Finally, a total of 108 patients underwent TNFi reduction and then followed maintaining therapy or a complete withdrawal, finished follow-up visit for at least 2 years (every 3 months in the first year and every 6 months in the second year), and completed at least baseline, 12-, and 24-month MRI scans of SIJ. Disease activity, such as Bath Ankylosing Spondylitis Disease Activity Index (BASDAI), Bath Ankylosing Spondylitis Functional Index (BASFI), and Ankylosing Spondylitis Disease Activity Score (ASDAS), clinical and laboratory parameters, and MRI of SIJ were collected and analyzed. Of the 108 patients, 63 patients who adopted a strategy of gradual reduction (reduce TNFi dose every 3 months) after achieving clinical remission or low disease activity and followed maintaining therapy of TNFi were defined as the tapering group. The rest 45 patients who completely stopped TNFi therapy after achieving clinical remission or low disease activity were referred to as the withdrawal group. The primary aim of our study was to investigate the relationship between the TNFi dose reduction and the disease relapse rate or the structural lesion progression of the SIJ, to obtain a reasonable and acceptable treatment strategy for some patients with axSpA who had to reduce or even stop TNFi for various reasons.

This study was approved by the Ethics Committee of Tongji Medical College of Huazhong University of Science & Technology (project identification code: 2020-S275). Clinical trial registration and ID number is ChiCTR2100043491. The retrieved data are de-identified and the requirement for informed consent was therefore waived.

### Interval Calculation

Each patient enrolled in the study was divided into five intervals, namely, 0–3, 3–6, 6–9, 9–12, and 12–24 months, based on the characteristics of the data available. Of the 108 patients, there were 540 intervals. We defined the response and relapse status in therapeutic intervals based on ASDAS-CRP (Supplementary Figure 1). The response status included two conditions, one of which was that the patients remained in remission (ASDAS-CRP <1.3) or low disease activity (ASDAS-CRP <2.1) for the entire interval, and the other of which was that the ASDAS-CRP score of the patients was changed from a higher level (ASDAS-CRP ≥ 2.1) to a lower level within the interval. Relapse status also included two conditions, one of which was that ASDAS-CRP score of the patients was changed from a lower level to a higher level (ASDAS-CRP ≥ 2.1) within the interval, the other of which was that patients remained high disease activity (ASDAS-CRP ≥ 2.1) unchanged for the entire interval. All 108 patients completed at least baseline, 12-, and 24-month MRI scans of SIJ. However, not all patients had MRI data at 3, 6, and 18 months. For the missing values, we used the linear interpolation method to impute based on the measured values before and after each follow-up point. Then we calculated the average dose quotient (DQ) (30), changes in fat metaplasia, erosion, backfill and the Spondyloarthritis Research Consortium of Canada (SPARCC) score, the status of bone marrow edema (SPARCC score increased or not), and the status of treatment response (response or relapse) for each interval.

### TNFi Reduction

In the tapering group, the TNFi dose was mainly characterized as followed: after the full dose of TNFi for 3–6 months to achieve disease remission or low disease activity, intervals of TNFi treatment were gradually prolonged, and each phase lasted for 3 months. In the first tapering phase, the dose was reduced to 66.7% or 50% of the full dose, in the second to 35% or 25%, and in the third to 16.7% or 12.5%. To quantify the dose reduction of TNFi, we used the term “DQ”, calculated as (actual dose/standard dose) × (standard dosing interval/actual dosing interval) × 100 according to Závadain et al. (30). For example, when etanercept is administered at 25 mg once a week instead of the full dose of twice a week at 25 mg, DQ = 25/25 × 3.5/7 × 100 = 50, which is 50% of the standard dosing regimen. In other words, a standard dose of etanercept (25 mg twice a week) is described as DQ100. Then during the first reduction, the dose of etanercept will be changed to 25 mg once a week, described as DQ50. During the second reduction, the dose of etanercept will be further changed to 25 mg once every 2 weeks, described as DQ25, and withdrawal described as DQ0. Other TNFis, such as adalimumab, infliximab, and golimumab, are reduced similarly (Supplementary Figure 3). The characteristics of the withdrawal group were mainly as follows: after the full dose of TNFi for 3–6 months to achieve low disease activity or disease remission, all patients in this group had rapidly reduced and stopped TNFi within 12 months. The average DQ of 3 months interval was calculated by adding the DQ at the beginning of the interval to the DQ at the end of the interval and then dividing by 2. The average DQ of 12–24 months interval was calculated as [(DQ at 12 months + DQ at 18 months)/2 + (DQ at 18 months + DQ at 24 months)/2]/2.

### Pelvic MRI

MRI-SIJ were obtained at least at baseline, 12, and 24 months and evaluated independently by two trained readers who were blinded for all clinical information and time order. The inflammation of SIJ was scored by the SPARCC scoring system using STIR images (33). Structural abnormalities, namely, erosion, fat metaplasia, and backfill, were evaluated by Sacroiliac Joint Structural Score (SSS) using T1-weighted images (34). The average of scores from the two readers was used as the final score.

### Data Collection and Statistical Analysis

The data of patients regarding demographics, age, gender, smoking status (ever vs. never), HLA-B27 positivity, disease duration, serum C-reactive protein (CRP), erythrocyte sedimentation rate (ESR), disease activity indices, such as BASDAI, BASFI, and ASDAS, were recorded at baseline. Disease activity (BASDAI, BASFI, and ASDAS), inflammatory biomarkers (CPR and ESR), and pelvic MRI data (SPARCC, fat metaplasia, erosion, and backfill) were collected at every follow-up visit from the electronic medical record system.

Numeric data were presented as mean (SE) or median [interquartile range, IQR], and categorical data were presented as percentages. The Student's *t*-test or Mann-Whitney U-test was used to compare the continuous variables and the Chi-square test or Fisher's exact test was used to compare the categorical data. All these descriptive data were analyzed with the R package “tableone”. To obtain an unbiased estimate of the effect of the average DQ value, we used inverse-probability-of-treatment weighting (IPTW) fitted to a marginal structural model (MSM) considering its ability to yield causal inference between the treatment exposure and outcome in the presence of time-dependent covariates that are also intermediate variables (35). Stabilized weights were calculated in IPTW with R package “ipw” (parameter “family” was set as “Gaussian” in this process), and the dominator as the estimated probability of treatment with different average DQ values was based on baseline covariates (gender, age, diagnosis, disease duration, smoking history, and positivity of HLA-B27), ASDAS-CRP value at the start of the follow-up interval. The numerator of weight was given by the estimated probability of different average DQ values treatment based on the baseline covariates aforementioned only. The correlation between IPTW weighted (or not) average DQ value and disease activity status or radiologic indices was realized with generalized estimating equation (GEE) via geeglm function in R package “geepack”. A receiver operator characteristic (ROC) curves were plotted with R package “pROC” and “WeightedROC”, and forest plots were realized with R package “forestplot”. All statistical analyses were performed with R version 4.0.3.

## Results

### Baseline Characteristics Between Two Groups

A total of 108 patients who underwent a dose reduction or complete withdrawal and followed up for at least 2 years were included in this study. Among them, there were 63 patients in the tapering group, 45 patients in the withdrawal group, and the proportions of patients with AS in the two groups were 57.1 and 60%, respectively, (*p* = 0.844). Baseline characteristics were similar between the two groups. All the patients had a high disease activity (BASDAI > 4 or ASDAS ≥ 2.1) at baseline, with the median (IQR) ASDAS-CRP of 3.45 (2.81, 4.21) in the tapering group and 3.14 (2.63, 3.96) in the withdrawal group. There was no difference in the disease activity and severity of structural damage at baseline (Table 1).

**Table 1:** Demographic and clinical characteristics of patients with axSpA at baseline.

		**Group**		***p* value**
		**Tapering (*n* = 63)**	**Withdrawal (*n* = 45)**	
Diagnosis (%)	nr-axSpA	27 (42.9)	18 (40.0)	0.844
	AS	36 (57.1)	27(60.0)	
Gender (%)	Female	21 (33.3)	14 (31.1)	0.838
	Male	42(66.7)	31 (68.9)	
Age (median [IQR])		28.00 [23.00, 33.00]	30.00 [25.00, 35.00]	0.145
Duration (median [IQR])		12.00 [3.00, 24.00]	13.00 [5.00, 24.00]	0.312
HLA-B27 (%)	–	10 (15.9)	11 (24.4)	0.327
	+	53 (84.1)	34 (75.6)	
Smoking (%)	–	38 (60.3)	26 (57.8)	0.844
	+	25 (39.7)	19 (42.2)	
SPARCC (median [IQR])		20.00 [13.00, 24.00]	18.00 [13.00, 25.00]	0.717
Fat metaplasia (median [IQR])		12.00 [7.00, 18.00]	12.00 [8.00, 16.00]	0.810
Erosion (median [IQR])		11.00 [7.00, 16.00]	11.00 [8.00, 17.00]	0.415
Backfill (median [IQR])		0.00 [0.00, 3.00]	0.00 [0.00, 3.00]	0.282
BASDAI (median [IQR])		4.00 [3.42, 4.50]	3.80 [3.05, 4.20]	0.124
BASFI (median [IQR])		1.70 [1.00, 2.70]	1.60 [0.75, 2.40]	0.373
ASDAS-CRP (median [IQR])		3.45 [2.81, 4.21]	3.14 [2.63, 3.96]	0.386
ASDAS-ESR (median [IQR])		3.45 [2.75, 4.17]	3.15 [2.67, 4.16]	0.477
CRP (median [IQR])		14.70 [8.40, 25.20]	18.90 [8.35, 29.35]	0.331
ESR (median [IQR])		24.00 [15.00, 40.00]	25.00 [13.50, 43.50]	0.935

*AS, ankylosing spondylitis; HLA, human leukocyte antigen; SPARCC, Spondyloarthritis Research consortium of Canada; BASDAI, Bath Ankylosing Spondylitis Disease Activity Index; BASFI, Bath Ankylosing Spondylitis Functional Index; ASDAS, Ankylosing Spondylitis Disease Activity Score; CRP, C-reactive protein; ESR, erythrocyte sedimentation rate; IQR, interquartile range*.

### Disease Activity and MRI Features at 12- and 24 Months Between Two Groups

At 12 months, BASDAI (0.40[0.00, 1.00] vs. 1.50[0.60, 2.10]; *p* < 0.001), BASFI (0.00[0.00, 0.40] vs. 0.50[0.00, 1.00]; *p* = 0.004), ASDAS-CRP (0.88[0.55, 1.62] vs. 1.83[1.29, 2.53]; *p* < 0.001), ASDAS-ESR (0.98[0.72, 1.52] vs. 1.77[1.23, 2.58]; *p* < 0.001), CRP (2.30[0.75, 5.25] vs. 5.21[1.70, 10.20]; *p* = 0.012), ESR (6.00[4.00, 14.50] vs. 10.00[6.00, 23.00]; *p* = 0.031), and SPARCC scores (1.00[0.00, 3.00] vs. 5.00[3.00, 7.00]; *p* < 0.001) in the tapering group were significantly lower than those in the withdrawal group. While the fat metaplasia, erosion, and backfill of SIJ were not statistically different between two groups. At 24 months, disease activity (BASDAI, BASFI, ASDAS-CRP, and ASDAS-ESR), inflammatory indicators (CRP and ESR), and SPARCC scores in the tapering group were still significantly lower than those in the withdrawal group. For the structural lesion, except fat metaplasia (21.00[15.00, 26.00] vs. 22.00[16.00, 25.00], *p* = 0.644), erosion (19.00[13.50, 24.50] vs. 22.00[16.00, 30.00]; *p* = 0.041), and backfill (4.00[3.00, 6.00] vs. 7.00[4.00, 8.00]; *p* < 0.001) were much lower in the tapering group (Table 2). These results suggested that patients in the tapering group had better control of disease activity and slower structural lesion progression of SIJ, especially at 24 months.

**Table 2:** Characteristic of disease activity and MRI features at months 12 and 24.

	**Group**		***p* value**
	**Tapering (n = 63)**	**Withdrawal (n = 45)**	
**Month 12**			
BASDAI (median [IQR])	0.40 [0.00, 1.00]	1.50 [0.60, 2.10]	**<0.001**
BASFI (median [IQR])	0.00 [0.00, 0.40]	0.50 [0.00, 1.00]	**0.004**
ASDAS-CRP (median [IQR])	0.88 [0.55, 1.62]	1.83 [1.29, 2.53]	**<0.001**
ASDAS-ESR (median [IQR])	0.98 [0.72, 1.52]	1.77 [1.23, 2.58]	**<0.001**
CRP (median [IQR])	2.30 [0.75, 5.25]	5.21 [1.70, 10.20]	**0.012**
ESR (median [IQR])	6.00 [4.00, 14.50]	10.00 [6.00, 23.00]	**0.031**
SPARCC (median [IQR])	1.00 [0.00, 3.00]	5.00 [3.00, 7.00]	**<0.001**
Fat metaplasia (median [IQR])	20.00 [14.00, 25.00]	20.00 [14.00, 24.00]	0.827
Erosion (median [IQR])	17.00 [11.50, 22.00]	18.00 [16.00, 24.00]	0.182
Backfill (median [IQR])	3.00 [0.00, 6.00]	4.00 [2.00, 6.00]	0.083
Relapse rate (%)	17.5	75.6	**<0.0001**
**Month 24**			
BASDAI (median [IQR])	1.20 [0.30, 1.80]	2.50 [1.50, 3.50]	**<0.001**
BASFI (median [IQR])	0.10 [0.00, 0.65]	0.90 [0.10, 1.50]	**0.003**
ASDAS-CRP (median [IQR])	1.57 [1.02, 2.38]	2.36 [1.77, 3.33]	**<0.001**
ASDAS-ESR (median [IQR])	1.48 [1.02, 2.00]	2.46 [1.82, 3.39]	**<0.001**
CRP (median [IQR])	5.20 [1.88, 9.29]	9.70 [3.47, 16.90]	**0.045**
ESR (median [IQR])	11.00 [5.50, 19.50]	20.00 [8.00, 38.00]	**0.007**
SPARCC (median [IQR])	3.00 [2.00, 5.00]	9.00 [8.00, 12.00]	**<0.001**
Fat metaplasia (median [IQR])	21.00 [15.00, 26.00]	22.00 [16.00, 25.00]	0.644
Erosion (median [IQR])	19.00 [13.50, 24.50]	22.00 [16.00, 30.00]	**0.041**
Backfill (median [IQR])	4.00 [3.00, 6.00]	7.00 [4.00, 8.00]	**<0.001**
Relapse rate (%)	69.8%	97.8%	**<0.001**

*SPARCC, Spondyloarthritis Research consortium of Canada; BASDAI, Bath Ankylosing Spondylitis Disease Activity Index; BASFI, Bath Ankylosing Spondylitis Functional Index; ASDAS, Ankylosing Spondylitis Disease Activity Score; CRP, C-reactive protein; ESR, erythrocyte sedimentation rate; IQR, interquartile range. Bolded values are statistically significant*.

**Figure 1:**
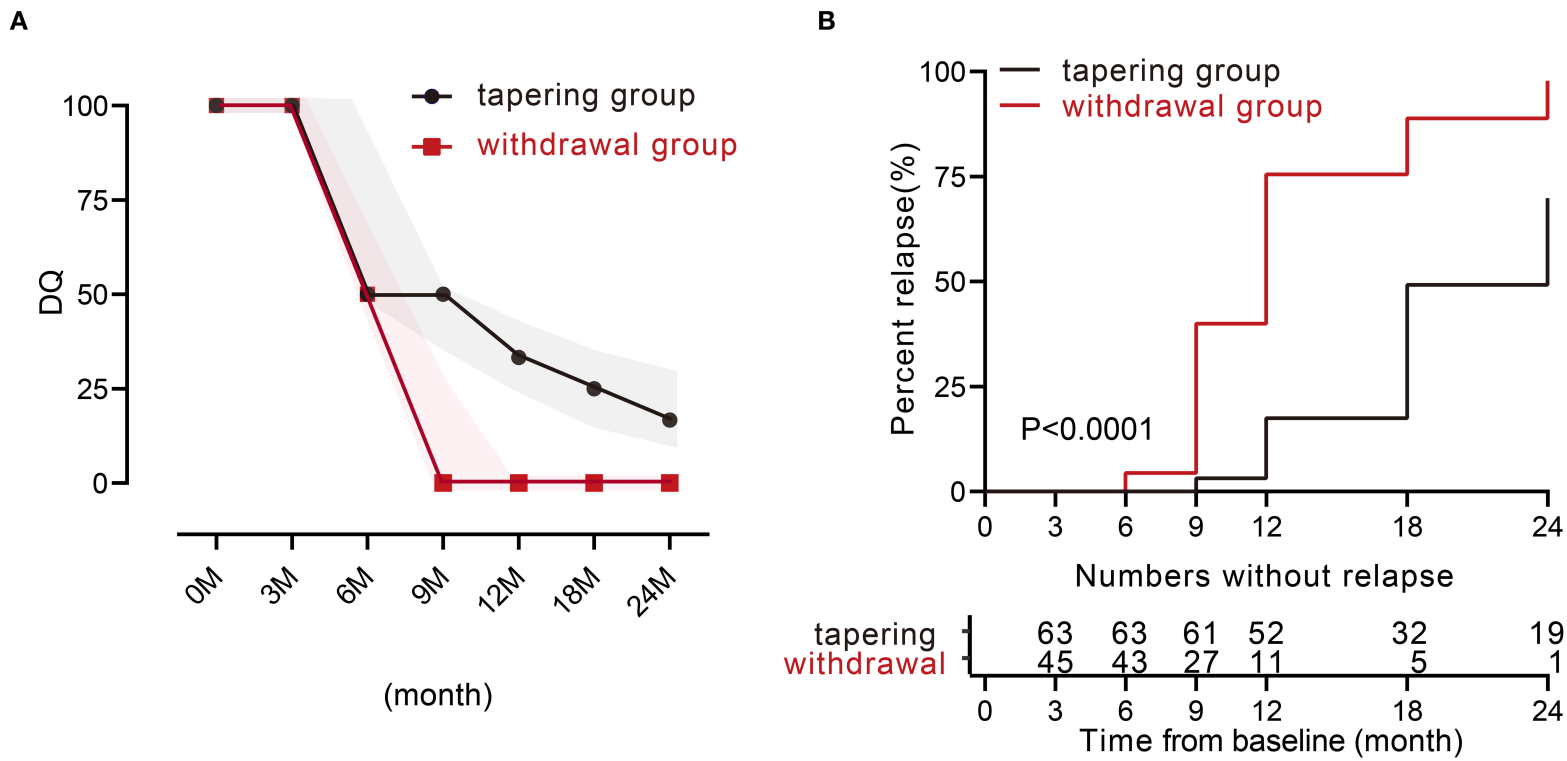
TNFi levels and characteristics of relapse during 24 months between the two groups. **(A)** Medians of dose quotient in the two groups during the 24 months. The shadow represents the interquartile range. **(B)** Relapse rate during the 24 months between the two groups (*p* < 0.0001). TNFi, tumor necrosis factor inhibitor.

We then analyzed the relapse rate and the DQ value of TNFi during 24 months. Only 11 patients experienced a relapse within the first year in the tapering group. The relapse rates in the tapering group and withdrawal group were 17.5% and 75.6% at 12 months (*p* < 0.001), and 69.8% and 97.8% at 24 months (*p* < 0.001). For the DQ values, at 0 and 3 months, both groups were 100 [100, 100]. At 6, 9, 12, 18, and 24 months, the tapering groups were 50 [50, 100], 50 [35, 50], 33.3 [25, 35], 25 [16.7, 33.3], and 16.7 [12.5, 33.3], respectively, while the withdrawal groups were 50 [0, 66.7], 25 [0, 25], 0 [0, 0], 0 [0, 0], and 0 [0, 0], respectively (Figure 1A). There were 19 patients (such as, 11 patients with AS) in the tapering group who did not experience a relapse throughout 24 months. Furthermore, the median time to relapse was much later in the tapering group [24 months (95% CI: 21–26)], compared with that in the withdrawal group [12 months (95% CI: 10–13)] (*p* < 0.0001, log-rank test; Figure 1B).

We further explored whether the response of patients with AS and non-axSpA to the TNFi reduction treatment was similar in each group. The results showed that there were no significant differences in disease activity, SPARCC score, and structural damage between the patients with AS and non-axSpA in each group at 12 months. There was also no significant difference in the tapering group at 24 months, while in the withdrawal group, patients with AS had more severe fat metaplasia at 24 months (data not shown).

### Relationship Between Average DQ and Structural Lesion Progression, Bone Marrow Edema, and Treatment Response

**Figure 2:**
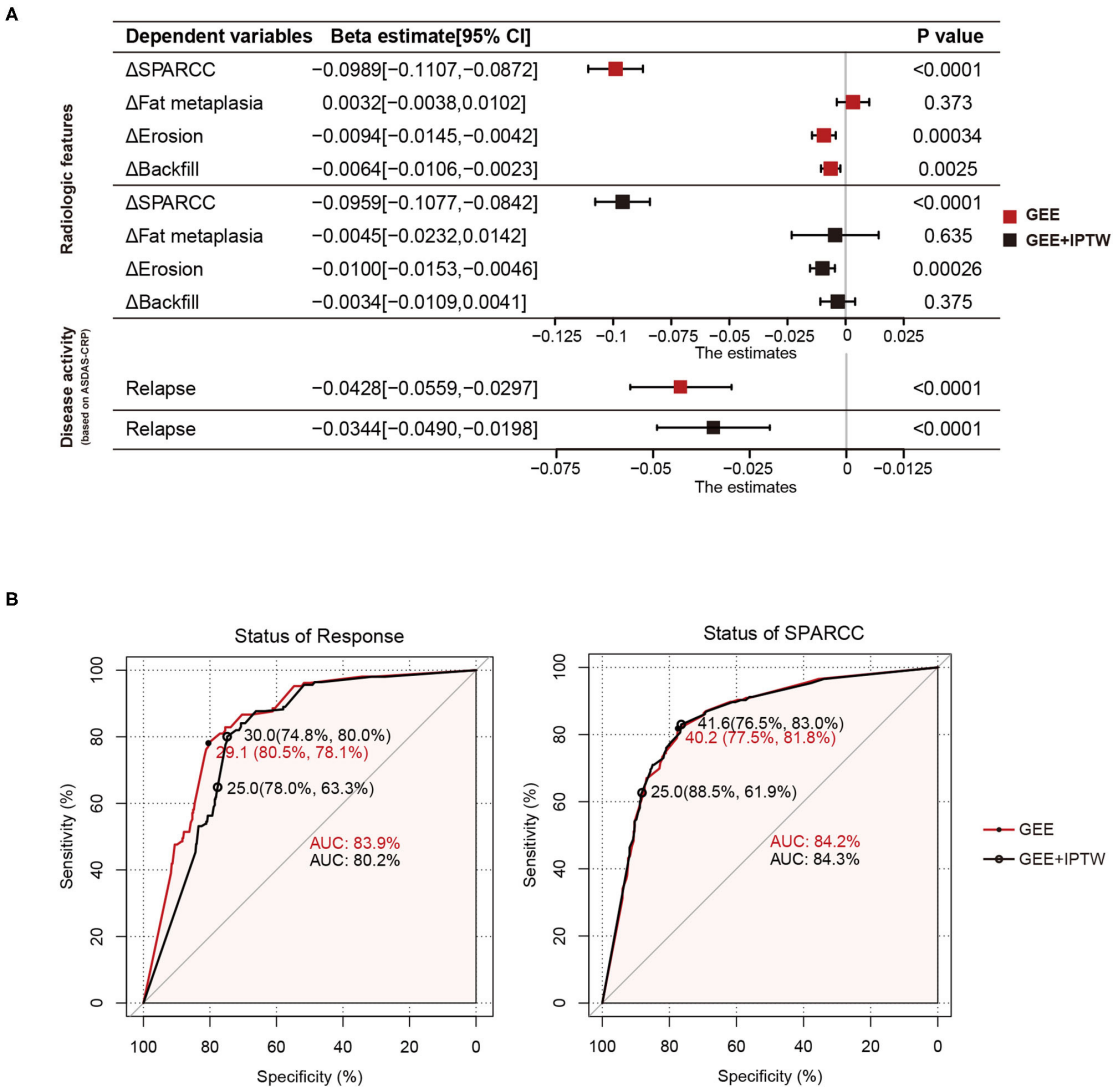
Average DQ values of 540 intervals from 108 patients with axSpA were fitted to relapse status and MRI features (change of SPARCC, fat metaplasia, erosion, and backfill score between the end and the start of the corresponding intervals) with GEE or GEE+IPTW methods. **(A)** Forest plots. **(B)** ROC curves of average DQ value in discriminating the status of treatment response (response or relapse) and status of SPARCC (SPARCC score increased or not). DQ, dose quotient; axSpA, axial spondyloarthritis; SPARCC, the Spondyloarthritis Research Consortium of Canada; GEE, generalized estimating equation; IPTW, inverse-probability-of-treatment weighting.

We have shown that backfill and erosion progress is slower in the tapering group at 24 months. To further investigate the unbiased effects between TNFi dose and structural progression, treatment response, or disease activity, we divided 2 years into five intervals and obtained 540 intervals from 108 patients. Average DQ, ΔSPARCC, Δfat metaplasia, Δerosion, Δbackfill, and the status of treatment were calculated for each interval. By using the GEE univariate model, we got the beta estimate of average DQ for ΔSPARCC was −0.0989 [−0.1107, −0.0872] (*p* < 0.0001), Δfat metaplasia was 0.0032 [−0.0038, 0.0102] (*p* = 0.373), Δerosion was −0.0094 [−0.0145, −0.0042] (*p* = 0.00034), Δbackfill was −0.0064 [−0.0106, −0.0023] (*p* = 0.0025), and the occurrence of relapse was −0.0428 [−0.0559, −0.0297] (*p* < 0.0001). We further adopted IPTW to adjust confounding factors. The adjusted beta estimates of average DQ for ΔSPARCC, Δfat metaplasia, Δerosion, Δbackfill, and the occurrence of relapse were −0.0959 [−0.1077, −0.0842] (*p* < 0.0001), −0.0045 [−0.0232, 0.0142] (*p* = 0.635), −0.0100 [−0.0153, −0.0046] (*p* = 0.00026), −0.0034 [−0.0109, 0.0041] (*p* = 0.375), and −0.0344 [−0.0490, −0.0198] (*p* < 0.0001) respectively (Figure 2A). These results suggested that Δerosion, ΔSPARCC, and the occurrence of relapse were negatively correlated with average DQ.

### ROC Curves to Evaluate Therapeutic Effects

Tapering or withdrawal of the TNFi could lead to relapse. To discriminate the treatment status of patients (response or relapse), we performed the ROC curve with two statistical models (GEE and GEE and IPTW). The area under the curve (AUC) was 83.9% (GEE) and 80.5% (GEE and IPTW). The optimal cut-off value was when the average DQ was 29.1 (80.5%, 78.1%) (GEE) and 30.0 (74.8%, 80%) (GEE and IPTW). Similarly, we also examined the treatment status of bone marrow edema (SPARCC score increased or not). The AUC was 84.2% (GEE) and 84.3% (GEE and IPTW). The optimal cut-off value was when the average DQ was 40.2 (77.5%, 81.8%) (GEE) and 41.6 (76.5%, 83%) (GEE and IPTW). These results indicated that at the average DQ 30, theoretically speaking, 80% of patients still achieved a good TNFi response, while at the average DQ 41.6, 83% of patients still obtained no progress of bone marrow edema (Figure 2B). However, considering operability and the convenience of clinical administration, we found that at average DQ 25, the status of treatment response (78%, 63.3%) and the status of bone marrow edema (88.5%, 61.9%) were also acceptable.

In addition, based on the feature of maintained DQ value, we found no patients relapsed among 16 patients who maintained DQ value over 25 (DQ > 25) in the tapering group at 24 months, while 44 of 47 (93.6%) patients who maintained DQ ≤ 25 in the tapering group relapsed during 24 months (Supplementary Figure 2).

### The Risk Factors for Relapse Before Average DQ Less Than 25

To further explore which factors were related to relapse before average DQ <25. We compared the baseline data for these 24 patients (relapse group) with the remaining 39 patients (response group) in the tapering group. Interestingly, we found that the positive rate of HLA-B27 was 100% in the relapse group and 74.4% in the response group (*p* = 0.010). In addition, the baseline fat metaplasia in the relapse group was 19.42 ± 1.92, significantly higher than that in the response group 9.62 ± 0.88 (*p* < 0.001). Other factors, such as gender, age, AS or not, smoking rate, SPARCC, erosion, backfill, BASDAI, BASFI, ASDAS-CRP, ASDAS-ESR, CRP, and ESR, were not statistically significant between these two groups (Table 3).

**Table 3:** Baseline data between patients relapsed or not before the average DQ <25 in the tapering group.

		**Group**		***p* value**
		**Relapse (*n* = 24)**	**Response (*n* = 39)**	
Diagnosis (%)	nr-axSpA	10 (41.7)	17 (43.6)	0.881
	AS	14 (59.3)	22 (56.4)	
Gender (%)	Female	8 (33.3)	13 (33.3)	1.000
	Male	16 (66.7)	26 (66.7)	
Age (median [IQR])		29.50 [23.00, 37.]	27.00 [23.00, 32.00]	0.220
Duration (median [IQR])		8.00 [3.00, 36.00]	12.00 [4.00, 24.00]	0.645
HLA.B27 (%)	−	0 (0.0)	10 (25.6)	**0.010**
	+	24 (100.0)	29 (74.4)	
Smoking (%)	−	15 (62.5)	23 (59.0)	0.781
	+	9 (37.5)	16 (41.0)	
SPARCC (median [IQR])		18.00 [11.25, 21.00]	22.00 [15.00, 25.00]	0.126
Fat metaplasia (mean ± SE)		19.42 ± 1.92	9.62 ± 0.88	**<0.001**
Erosion (mean ± SE)		10.00 ± 1.42	12.21 ± 0.89	0.171
Backfill (median [IQR])		0.00 [0.00, 2.75]	0.00 [0.00, 3.00]	0.853
BASDAI (median [IQR])		3.95 [3.22, 4.87]	4.00 [3.50, 4.50]	0.921
BASFI (median [IQR])		1.80 [1.05, 3.25]	1.70 [0.90, 2.50]	0.859
ASDAS-CRP (median [IQR])		3.48 [2.38, 3.99]	3.45 [2.85, 4.23]	0.436
ASDAS-ESR (median [IQR])		3.24 [2.46, 4.00]	3.46 [2.77, 4.46]	0.315
CRP (median [IQR])		13.65 [5.39, 28.94]	14.70 [9.26, 23.67]	0.921
ESR (median [IQR])		21.50 [10.50, 44.25]	25.00 [17.00, 38.00]	0.552

*AS, ankylosing spondylitis; HLA, human leukocyte antigen; SPARCC, Spondyloarthritis Research consortium of Canada; BASDAI, Bath Ankylosing Spondylitis Disease Activity Index; BASFI, Bath Ankylosing Spondylitis Functional Index; ASDAS, Ankylosing Spondylitis Disease Activity Score; CRP, C-reactive protein; ESR, erythrocyte sedimentation rate; IQR, interquartile range; SE, standard error. Bolded values are statistically significant*.

## Discussion

In the clinic, some patients have to opt for dose reduction or withdrawal because of intolerance to full-standard TNFi, risk of potential infection, unaffordability to high expense, intolerability for long-term subcutaneous injection, or multiple reasons. EULAR recently recommended that tapering TNFi can be considered for patients who have achieved sustained remission. However, there is no unified standard yet. To explore a referable dose reduction strategy, we retrospectively examined the effects of different TNFi dose-reduction strategies on patients with axSpA. Firstly, rapid dose reduction then complete withdrawal after clinical symptom relief was not recommended. Secondly, our study provided a reference strategy that gradually tapering followed by maintaining the average DQ over 30 for patients who need TNFi reduction, or over 25 for patients with HLA-B27 negative and non-severe fat metaplasia at baseline were acceptable.

Several studies have explored the efficacy of tapering strategies in patients with axSpA (26–32). Landewé et al. found that after achieving sustained remission at 48 weeks, a half dose of certolizumab pegol could maintain 79% of patients flare-free for the next 48 weeks in patients with early axSpA, which was comparable to 83.7% in the full-dose group. Gratacós et al. claimed that after achieving clinical remission for ≥6 months, 81.3% of patients in the reduced dose arm maintained low disease activity in the next year, which is comparable to that of patients in the full-dose arm (83.8%). In our study, the disease activity was much lower in the tapering group compared with the withdrawal group at months 12 and 24. At month 12, 95.2% (60/63) of the patients in the tapering group reduced the TNFi dose to 50% (12 patients) or <50% (48 patients). At this time point, 82.5% (52/63) of the patients were still without relapse in this group. However, with further reduction, the relapse rate and the relapse risk increased significantly. In addition, we also found that the cut-off value of the average DQ was 30 for maintaining a relatively good treatment response. Therefore, TNFi tapering is an alternative option for those who cannot use full-dose TNFi due to various reasons, but the maintaining dose of TNFi should not be reduced to a level below average DQ 30. In addition, rapid dose reduction then complete withdrawal after clinical symptom relief was not recommended. However, in the clinic, the average DQ 30 is not operable and convenient for clinical administration, so we further analyzed the average DQ 25 and found the treatment effects were acceptable. In addition, the relapse rate of patients with DQ >25 was significantly lower than patients with DQ ≤ 25 at 24 months.

Many studies have focused on the efficacy of TNFi reduction therapy, but few studies paid attention to the risk factors of disease relapse during the reduction process. Almirall et al. found that a shorter duration of remission before dose reduction, shorter duration of TNFi treatment, and shorter disease duration were the risk factors of relapse (36). In our study, we found that patients with HLA-B27 positive or more severe fat metaplasia at baseline were more likely to relapse before the average DQ was <25. Previous studies have reported that the presence of HLA-B27 in patients with AS is correlated with higher disease activity and poor functional status (37–39). In addition, evidence showed that HLA-B27 influences disease activity by a pathway not involving TNF-α (40). This may be the reason why patients with HLA-B27 positive had a higher risk of relapse during dose reduction of TNFi. Studies showed that fat metaplasia appeared to be a feature of tissue response after inflammation resolution, and it was a risk factor for the development of syndesmophytes and ankylosis (41–43). Therefore, reducing the average DQ to <25 was not recommended especially for those patients with HLA-B27 positive or severe fat metaplasia.

Only a few studies investigated the effect of TNFi on SIJ of patients with axSpA. Almirall et al. found no significant progress in the SIJ in a cohort of patients with nr-axSpA treated with TNF-α blockers for 2 years (44). Another study of the RAPID-axSpA phase III randomized trial found that patients with axSpA treated with TNFi did not show significant SIJ progression after 4 years (45). However, these two studies lacked matched controls. Dougados et al. assessed the changes of SIJ in patients with recent onset axSpA receiving etanercept for 2 years and found a slower progression of SIJ compared with patients not receiving TNFi (20). In our study, we also found that the tapering strategy had a slower progression of erosion in SIJ. Another retrospective cohort study observed the effects of TNFi reduction on hip arthritis, showing that the acute inflammatory changes in the full-dose group and tapering group were equivalent (28). In our research, we used MRI to observe inflammation for SIJ and suggested that tapering TNFi could better control bone marrow edema compared with the complete withdrawal of TNFi. What is more, higher doses of TNFi treatment resulted in less severe bone marrow edema and slower erosion progression of the SIJ.

This study has some limitations to be considered. Firstly, the sample size of each group was relatively small. Secondly, because it was a retrospective study, it was impossible to ensure that the tapering strategy for each patient is strictly uniform, but we used DQ and average DQ to minimize this bias, according to a study published by Závadain et al. (30). Our study firstly explored the correlation between the average DQ and structural lesion progression of SIJ in the tapering therapy and explored a referable reduction strategy in the clinic for those patients who need TNFi reduction because of various reasons. In conclusion, we suggest that gradually tapering followed by maintaining the average DQ over 30 or even 25 is an acceptable strategy for patients who need TNFi reduction. However, we also indicate that a higher dose of TNFi is associated with a slower erosion progression of SIJ. Therefore, in clinical practice, we need to adopt appropriate treatment strategies according to the actual situation of the patients. Although future randomized controlled investigations with a larger sample size are needed to provide more information, this retrospective cohort study provided a referable value on the therapeutic efficacy of this TNFi tapering strategy for patients with axSpA.

## Data Availability Statement

The raw data supporting the conclusions of this article will be made available by the authors, without undue reservation.

## Ethics Statement

The studies involving human participants were reviewed and approved by Tongji Medical College of Huazhong University of Science and Technology (project identification code: 2020-S275). Written informed consent for participation was not required for this study in accordance with the national legislation and the institutional requirements.

## Author Contributions

LD and MW designed the study. QM and YD analyzed the data and wrote the paper. CY, JZ, and SC contributed to the interpretation of the data. All authors revised the paper and approved the final manuscript.

## Funding

This work was supported by grants from the National Natural Science Foundation of China (No. 81771754) and the Tongji Hospital Clinical Research Flagship Program (No. 2019CR206).

## Conflict of Interest

The authors declare that the research was conducted in the absence of any commercial or financial relationships that could be construed as a potential conflict of interest.

## Publisher's Note

All claims expressed in this article are solely those of the authors and do not necessarily represent those of their affiliated organizations, or those of the publisher, the editors and the reviewers. Any product that may be evaluated in this article, or claim that may be made by its manufacturer, is not guaranteed or endorsed by the publisher.
