# Supplementary material for: Structural Lesion Progression of the Sacroiliac Joint and Clinical Features in axSpA During TNFi Reduction: A Retrospective Cohort Study
Source: Front Med (Lausanne). 2021 Dec 7;8:781088. doi: 10.3389/fmed.2021.781088 (PMC8688735; doi:10.3389/fmed.2021.781088)
Supplement: Supplementary file 1 [file Data_Sheet_1.docx]

Supplementary Figure S1. The definitions of response and relapse are based on ASDAS-CRP.


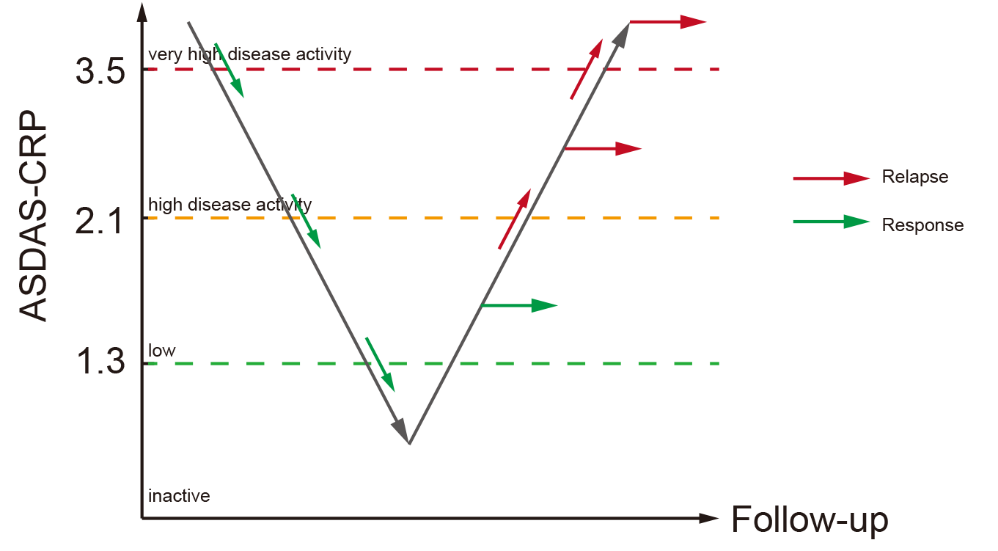


The green arrow represents the status of response, while the red arrow represents the status of relapse.

Supplementary Figure S2. The feature between the maintained DQ value and relapse.


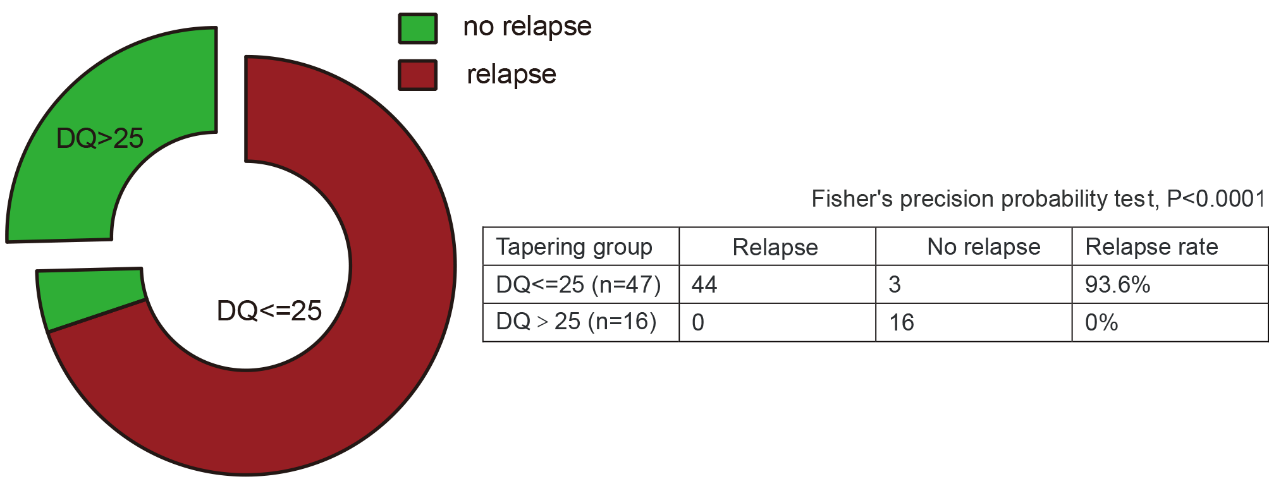


 No patients relapsed among 16 patients who maintained DQ value over 25 (DQ>25) in the tapering group at 24 months, while 44 of 47 (93.6%) patients who maintained DQ≤25 in the tapering group relapsed during 24 months.

Supplementary figure S3. A diagram of dose reduction or discontinuation (Take etanercept as an example).


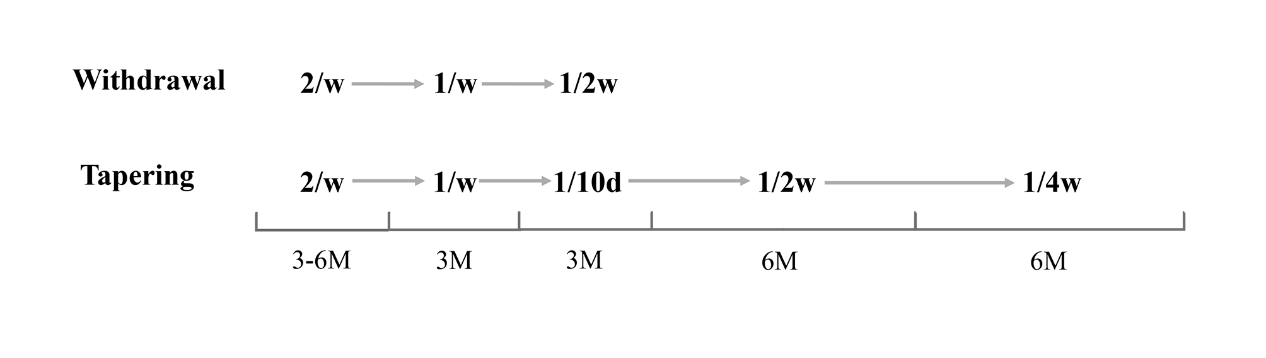


In the withdrawal group, when DQ≤50 (1/w), patients discontinued the TNFi or continued to reduce the dose, and all patients discontinued TNFi within 1 year. In the tapering group, when DQ≤35 (1/10d), patients continued to extend the interval or maintain the TNFi dose. W, week; 2/w, twice a week; d, day; 1/10d, once every 10 days; M, month.
